# Rare Cerebral Location of a Left Lateral Ventricle Solitary Fibrous Tumor (SFT): A Case Report With Literature Review

**DOI:** 10.7759/cureus.65215

**Published:** 2024-07-23

**Authors:** Ayoub Abdellah, Mohammed Lhamlili, Hamid Khay, Mounir Rghioui, Mohamed Khoulali, Faycal Moufid

**Affiliations:** 1 Department of Neurosurgery, University Hospital Mohammed Vl, Faculty of Medicine and Pharmacy of Oujda, University Mohammed First, Oujda, MAR; 2 Department of Neurosurgery, Mohammed Vl International University Hospital, Mohammed VI University of Sciences and Health (UM6SS), Casablanca, MAR

**Keywords:** brain mri, brain, intracranial hypertension, emergency neurosurgery, solitary fibrous tumor (sft)

## Abstract

Solitary fibrous tumors (SFTs) are rare mesenchymal neoplasms that can occur intraventricularly, presenting diagnostic and management challenges. We describe a case of a 21-year-old male with no significant medical history who presented with intermittent headaches and vomiting, progressing to continuous symptoms. Neurological examination was unremarkable. Brain MRI revealed an isointense lesion in the occipital horn of the left lateral ventricle, diagnosed as an SFT. Surgical excision via a transcortical approach was successful, followed by postoperative radiotherapy. This case highlights the complexities in diagnosing and treating intraventricular SFTs, emphasizing the need for comprehensive evaluation and multimodal management strategies.

## Introduction

Solitary fibrous tumors (SFTs) are rare mesenchymal neoplasms that can occur in various anatomical locations, including the central nervous system. Intraventricular SFTs are particularly uncommon, with distinct clinical and radiological characteristics that necessitate meticulous differential diagnosis from other intraventricular tumors such as meningiomas, metastases, high-grade gliomas, and subependymomas. Accurate diagnosis is critical as it influences the therapeutic management and prognosis [1-7]. Here, we present a case of a 21-year-old male who presented with intermittent headaches and vomiting, later diagnosed with an intraventricular solitary fibrous tumor. This case highlights the importance of thorough clinical, radiological, and histopathological evaluation in the management of intraventricular tumors.

## Case presentation

We report the case of a 21-year-old male with no significant medical history who presented with a three-week history of intermittent headache and vomiting. Over the following days, his symptoms worsened, characterized by continuous headache and vomiting. There were no signs of facial asymmetry. He had no family history of neurological disorders and no history of substance abuse.

On clinical examination, the patient was conscious and well-oriented, with stable vital signs. Neurologically, he displayed normal gait and posture, normal muscle tone, and preserved global and segmental muscle strength without any deficits. Osteotendinous reflexes were symmetrical and of normal amplitude, and cranial nerve examination was intact. Babinski and cerebellar signs were absent, and systemic examination showed no abnormalities.

**Figure 1:**
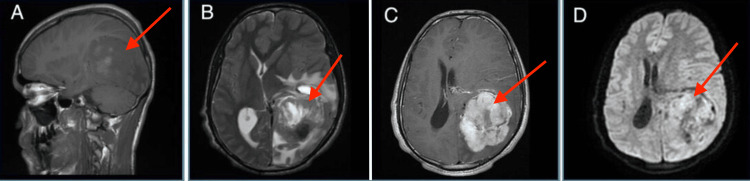
Brain MRI showing a 5 x 5 x 4 cm multilobulated mass in the occipital horn of the left lateral ventricle with intense contrast enhancement, perilesional edema, and mass effect, but no dural tail sign, with a central necrotic area (red arrows). (A) Sagittal T1 sequence showing hypointense to isointense T1 signal. (B) T2 sequence showing a heterogeneous lesion with both hypo- and hyperintense T2 signals. (C) Axial T1 sequence with gadolinium contrast showing intense enhancement with mass effect and midline shift. (D) DWI sequence showing a heterogeneous lesion with a low ADC index. DWI: Diffusion-weighted imaging, ADC: Apparent diffusion coefficient.

Laboratory assessments, including a complete blood count with differentials and thyroid-stimulating hormone levels, were without abnormalities. Serological tests for hepatitis viruses, HIV, and syphilis were negative. A brain MRI revealed a lesion in the occipital horn of the left lateral ventricle. The minimum apparent diffusion coefficient (ADC) was 0.70 × 10⁻³ mm²/s, suggesting a solitary fibrous tumor (SFT) (Figure 1).

**Figure 2:**
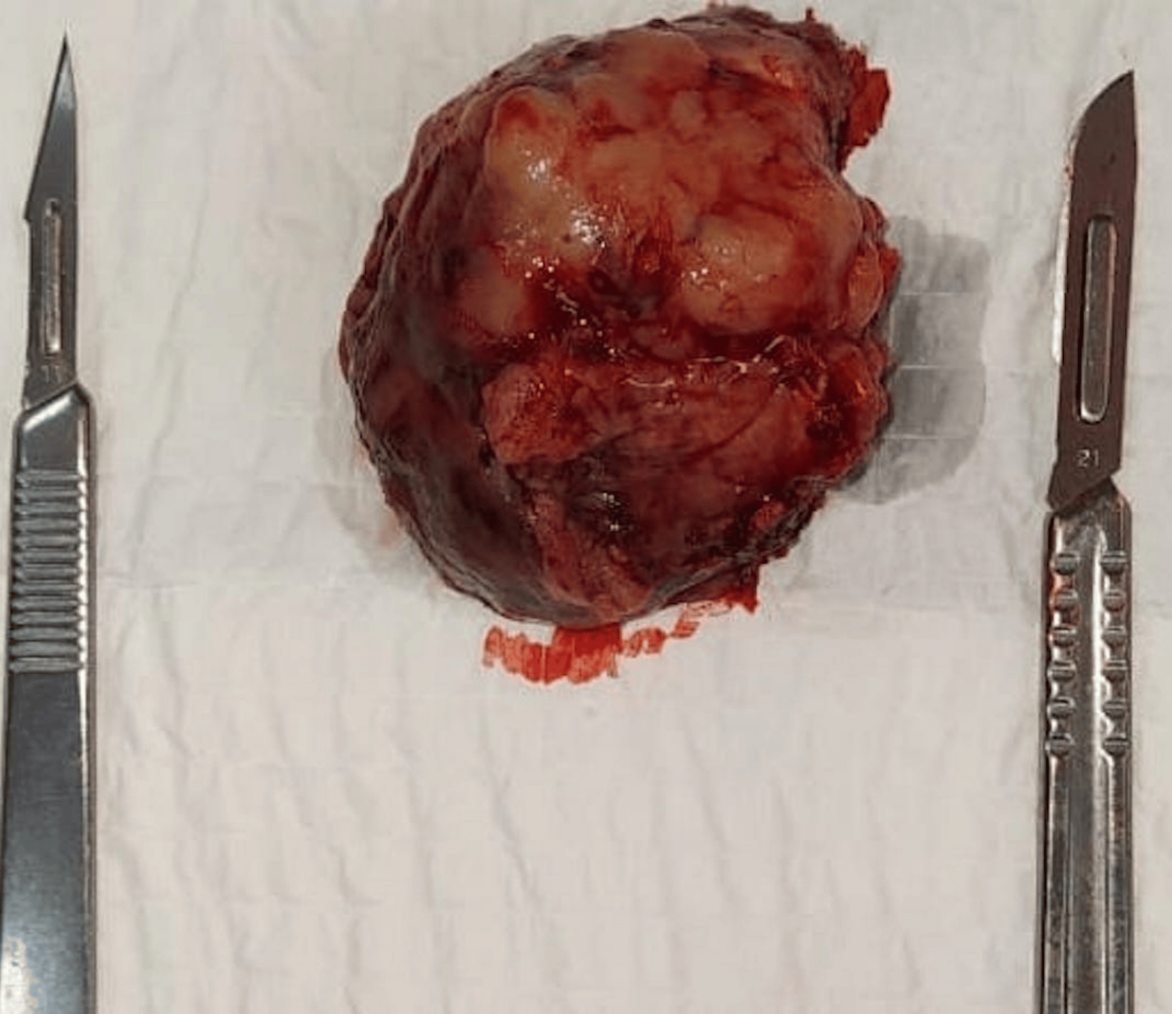
Preoperative image of the excised tumor.

**Figure 3:**
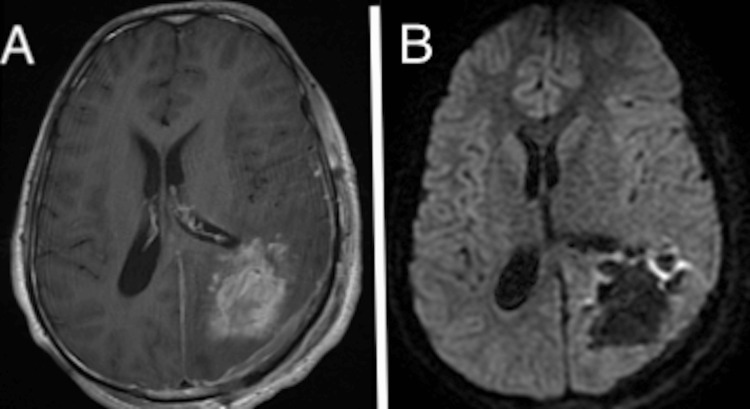
Postoperative brain MRI showing total resection with the disappearance of the mass effect. (A) Axial T1 postcontrast and (B) DWI. DWI: Diffusion-weighted imaging.

The neurosurgery team opted for tumor excision following an embolization procedure (Figure 2). Intraoperatively, a transcortical occipital approach was employed, achieving a total resection of the lesion via an occipital arciform incision with a centered bone flap, followed by an X-shaped dural opening. A postoperative MRI was performed to confirm the resection (Figure 3).

**Figure 4:**
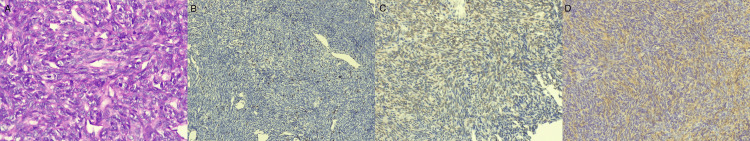
A tumor resection specimen weighing 50 g and measuring 5 x 5 x 4 cm, exhibits a firm consistency and a whitish-gray color with some areas of hemorrhagic changes. (A) Histopathological 40x magnification image of oblong cells with ovoid, hyperchromatic nuclei. (B) Anti-Ki67 antibody staining at 10x magnification, estimated labeling of 10 to 15% of tumor cells. (C) Anti-Stat 6 antibody staining at 40x magnification, negative. (D) Anti-CD34 antibody staining at 10x magnification, showing positive tumor cells: morphological and immunohistochemical aspects in favor of a solitary fibrous tumor, grade III according to the 2016 WHO classification.

A pathological diagnosis of the tumor was performed and confirmed the diagnosis of SFT, grade III according to the 2016 WHO classification (Figure 4).

Following the surgery, the case was reviewed by the oncology team, who recommended postoperative radiotherapy for the patient. The patient was clinically monitored after one month, then after three months, and underwent a brain MRI in the sixth month. 

## Discussion

For intraventricular tumors, differential diagnosis must consider the patient's age and the lesion's location. Potential diagnoses in this case include meningioma, metastasis, high-grade glioma, subependymoma, choroid plexus papilloma, ependymoma, subependymal giant cell astrocytoma (SGCA), and central neurocytoma [1-3]. Differentiating SFTs from meningiomas is essential due to their similar imaging feature, yet they have different treatment strategies and prognoses. Risk factors include the patient's age, tumor location, and radiological features [4].

Meningioma is the most common extra-parenchymal and supratentorial tumor in adults. Intraventricular meningiomas are rare, comprising only 0.7% of all meningiomas, yet they are among the most frequent intraventricular tumors in adults [3]. Radiological characteristics of meningiomas include well-defined masses on non-contrast CT scans, a slightly hyperdense appearance compared to the parenchyma, and potential calcifications [3].

In the brain, SFTs exhibit distinct characteristics. On T1-weighted sequences, they appear as isointense masses, and on T2-weighted sequences, they typically appear hyperintense, although they may also present as heterogeneous lesions with both hyperintense and hypointense areas. After gadolinium administration, SFTs generally show homogeneous and intense enhancement, although heterogeneous enhancement has also been reported [4].

To differentiate SFTs from meningiomas on MRI, consider signs such as lobulated borders, rare calcifications, tight attachment to the base, presence of the flow void sign, rare hyperostoses of the adjacent bone, and also peritumoral brain edema [1,2,5]. SFTs tend to cause bone erosion rather than hyperostosis. Chiechi et al. found that nearly 60% of SFT cases exhibited bone erosion, with none showing bone hyperostosis [5]. The dural tail sign, common in meningiomas (observed in 52% to 78% of cases), is less frequent in SFTs. Pang et al. noted the dural tail sign in only 1 out of 15 SFT cases [6,7].

Indicators of malignancy in SFTs on high-resolution MRI include irregular tumor shape, indistinct margins, lobulated necrotic borders, and bone destruction [8,9]. The ADC in the diffusion-weighted imaging (DWI) sequence can help distinguish between meningiomas and different grades of SFTs. With a threshold value of 1.15×10⁻³ mm²/s, the ADC indicator has a sensitivity of 75% and specificity of 60.42% [10-13]. Meningiomas typically have higher ADC values compared to even grade 1 SFT/hemangiopericytomas (HPC) [12,13]. Mama et al. reported that grade II SFT/HPCs had higher ADC values than grade III SFT/HPCs (1.26-1.50 × 10⁻³ mm²/s versus 0.638-0.833 × 10⁻³ mm²/s, respectively) [14].

A literature review of 21 cases of ventricular SFTs revealed a mean age of 47.33 years (range 9-69) and a gender distribution of 13 males to eight females. The tumor was predominantly located in the lateral ventricles (12 out of 21 cases), followed by the 4th ventricle (eight out of 21 cases), and only one case was found in the 3rd ventricle [15]. The preferred surgical approach was the telovelar approach (eight out of 21 cases), followed by the transcortical approach (seven out of 21 cases), and the anterior transcallosal approach (three out of 21 cases). Notably, two authors did not specify their surgical approach [15]. In our case, the patient was a 21-year-old male with a lesion located in the occipital horn of the left ventricle. The surgical approach used was transcortical, utilizing neuronavigation, a microscope, and the Doro retractor system.

The management of SFTs involves a multimodal treatment approach combining complete surgical resection followed by postoperative radiotherapy. SFTs have a high risk of local and locoregional recurrence, ranging from 38% to 91% depending on the initial treatment, with most recurrences occurring at the site of initial resection [7]. Currently, there is no consensus on SFT management, particularly in recurrent cases [7]. Surgical resection as the first-line treatment significantly impacts recurrence-free survival and local control. Total tumor resection (TTR) is crucial for improving outcomes [14].

Numerous studies have examined the impact of postoperative radiotherapy (PORT) on recurrence and survival in meningeal SFTs/HPCs, with mixed results. Some studies demonstrate the effectiveness of PORT for local control, progression-free survival (PFS), and even overall survival (OS) [16-21]. However, Xiao et al. and Rutkowski et al. did not find an association between PORT and OS or PFS [7,21]. In our case, the patient received PORT of 50 Gy. Preoperative embolization is recommended as a safe method to reduce tumor size and the risk of blood loss, and it can be performed with the aim of facilitating radiotherapy [7].

Chemotherapy has been explored for treating recurrent tumors, whether metastatic or not when local treatment is not feasible. However, metastatic or recurrent meningeal SFTs/HPCs are generally considered relatively insensitive to chemotherapy [21]. Targeted therapies, including anti-angiogenics, interferon alpha (IFN-α), and tyrosine kinase inhibitors, are based on the overexpression of IGF2 and the Akt/mTOR signaling pathway. These treatments have shown stabilization and better tumor responses compared to chemotherapy in small cohorts [16-20].

SFTs are often more aggressive, with a higher risk of local recurrence and metastasis, reaching rates up to 20%, necessitating closer and longer post-therapeutic follow-up [6]. While no consensus protocol exists for follow-up evaluations of these rare tumors, lifelong monitoring is recommended due to their unpredictable behavior. A suggested protocol includes regular clinical follow-ups, brain MRIs every six months to one year, and chest X-rays to detect pulmonary metastases. Complaints of bone pain should prompt further investigation for bone metastases [4].

Given the aggressive nature of intracranial SFTs/HPCs, it is plausible that intraventricular lesions can spread within the neuraxis via cerebrospinal fluid. However, documented cases are rare. One case involved a 19-year-old man who developed extensive spinal metastases five months after radiotherapy and local adjuvant irradiation, likely originating from initial tumor hemorrhage [13]. Although long-term follow-up of intraventricular SFT/HPC patients is uncommon, some cases have been monitored for over five years, with the longest follow-up being 21.5 years, showing no signs of local recurrence or distant metastasis [4].

## Conclusions

In conclusion, solitary fibrous tumors within the ventricles, though rare, should be considered in the differential diagnosis of intraventricular masses, especially in young patients presenting with symptoms such as headaches and vomiting. This case emphasizes the need for a comprehensive diagnostic work-up, including advanced imaging techniques and histopathological confirmation, to differentiate solitary fibrous tumors (SFTs) from other intraventricular tumors. Multimodal treatment approaches, primarily surgical resection followed by postoperative radiotherapy, are essential for effective management and improving patient outcomes. Given the aggressive nature and potential for recurrence and metastasis, long-term follow-up is crucial for patients diagnosed with intraventricular SFTs.
